# A fair experimental comparison of neural network architectures for latent representations of multi-omics for drug response prediction

**DOI:** 10.1186/s12859-023-05166-7

**Published:** 2023-02-14

**Authors:** Tony Hauptmann, Stefan Kramer

**Affiliations:** Department of Computer Science, Johannes Gutenberg University Mainz, Mainz, Germany

**Keywords:** Deep learning, Multi-omics, Machine learning, Drug response, Neural network, Autoencoder

## Abstract

**Background:**

Recent years have seen a surge of novel neural network architectures for the integration of multi-omics data for prediction. Most of the architectures include either encoders alone or encoders and decoders, i.e., autoencoders of various sorts, to transform multi-omics data into latent representations. One important parameter is the depth of integration: the point at which the latent representations are computed or merged, which can be either early, intermediate, or late. The literature on integration methods is growing steadily, however, close to nothing is known about the relative performance of these methods under fair experimental conditions and under consideration of different use cases.

**Results:**

We developed a comparison framework that trains and optimizes multi-omics integration methods under equal conditions. We incorporated early integration, PCA and four recently published deep learning methods: MOLI, Super.FELT, OmiEmbed, and MOMA. Further, we devised a novel method, Omics Stacking, that combines the advantages of intermediate and late integration. Experiments were conducted on a public drug response data set with multiple omics data (somatic point mutations, somatic copy number profiles and gene expression profiles) that was obtained from cell lines, patient-derived xenografts, and patient samples. Our experiments confirmed that early integration has the lowest predictive performance. Overall, architectures that integrate triplet loss achieved the best results. Statistical differences can, overall, rarely be observed, however, in terms of the average ranks of methods, Super.FELT is consistently performing best in a cross-validation setting and Omics Stacking best in an external test set setting.

**Conclusions:**

We recommend researchers to follow fair comparison protocols, as suggested in the paper. When faced with a new data set, Super.FELT is a good option in the cross-validation setting as well as Omics Stacking in the external test set setting. Statistical significances are hardly observable, despite trends in the algorithms’ rankings. Future work on refined methods for transfer learning tailored for this domain may improve the situation for external test sets. The source code of all experiments is available under https://github.com/kramerlab/Multi-Omics_analysis

**Supplementary Information:**

The online version contains supplementary material available at 10.1186/s12859-023-05166-7.

## Background

Data analysis in the life sciences often involves the integration of data from multiple modalities or views. Integration is necessary to obtain models with improved predictive performance or explanatory power. One currently popular approach to integrate multiple views is to take advantage of latent representations as computed by neural network architectures. Views are usually defined as (potentially very large) groups of variables that originate from one measurement technology. In bioinformatics and computational biology, views often originate from different omics platforms, e.g., from genomics, transcriptomics, proteomics, and so forth.

Frequently, views from different omics platforms are collected by different research groups, which then evaluate them separately. This independent analysis of views can lead to inconsistent conclusions, which are hard to interpret when combined [1]. The problem is aggravated with data about complex diseases like cancer, which are impossible to understand by a single view only [2].

Each view provides distinct, complementary information about a phenomenon of interest, and integrating them can lead to an improved understanding of the underlying biology [3]. Previous studies compared single-omics and multi-omics approaches and indicated that an increased predictive performance for cancer classification and survival analysis is achieved by using multi-omics approaches [1, 2, 4, 5].

Given several views, there exist mainly three different paradigms of how to integrate them: early, late, and intermediate integration. They depend on the point at which information of distinct views is combined [6].

In *early integration*, the view’s features are concatenated into a single representation and analyzed with traditional methods. This has the advantage of being simple, but the disadvantage of creating a higher-dimensional and sparse feature space. Early integration methods assume a high dependency between omics. Furthermore, they are not suitable for combining non-tabular views as for example visual data or text.

*Late integration*, on the other hand, combines the results of models, which are trained independently on a single view, to obtain the final prediction. It models interaction between views only by weighting the single-view results differently. Late integration allows the combination of heterogeneous views, as only the output of a model is essential and view-specific models can be trained.

Whereas early integration assumes a high dependency between views and late integration a low dependency [6], *intermediate integration* represents a trade-off between the both. In intermediate integration, the views are first transformed to a lower dimensional representation with a suitable encoder. Only then the transformed representations are concatenated and evaluated.

The encoding of a view into the latent feature space is widely performed with neural networks. The neural network architectures include either just encoders or encoders and decoders, as in autoencoder-type architectures. A myriad of architectures is possible for the integration of multi-omics data: encoders only, encoders-decoders (autoencoders of various sorts), integration either early (already for the computation of a joint latent representation), intermediate (concatenating latent representations following their computation), or late (combining the results of individual latent representations) [7], and using different loss functions.

As the literature on the topic is growing, one would expect that a more recent publication would improve upon a previous publication in terms of performance, or, at least, that a specific use case with superior performance has been identified then. However, as it turns out, various approaches have been optimized and tested with different sets of hyperparameters (some of them fixed, some of them optimized), with different values for hyperparameters, and with different test protocols. Further, performance can be very different for cross-validation (assuming a similar distribution of data for each test set) and so-called external test sets. Thus, it is at this point far from clear which method performs best overall, and, specifically, which method is to be preferred in which setting, e.g., in a cross-validation-like setting or with an external test set from an unknown distribution.

In this paper, we level the playing field, establish a uniform, basic protocol for various existing methods, and test the methods with a state-of-art statistical method for the comparison of machine learning algorithms [8]. As a side product, we derive a method that integrates intermediate and late integration and fares well in settings where the predictive performance on external test sets is favored.

We study the prediction performance of the various algorithms on data sets for drug response prediction, which have been used widely in the literature in past few years [4]. The ultimate goal of such studies is to detect drug response biomarkers, which would help to develop personalized treatments of patients and improve clinical outcomes [9]. Drug screening studies on large patient cohorts are rarely performed, because it is ethically not feasible to change the chemotherapeutic regime and to cause a suboptimal therapy [9, 10]. On the other hand, large-scale drug-screening efforts using human cancer cell line models have begun to establish a collection of gene-drug associations and have uncovered potential molecular markers predictive of therapeutic responses [11]. A critical challenge that remains is the clinical utility of the results, i.e., the translatability from *in vitro* to *in vivo* [4].

In summary, the contributions of this paper are as follows:

- A fair comparison of recent deep multi-omics integration algorithms for drug response prediction,
- A new combined intermediate and late architecture for multi-omics integration, and
- A thorough validation study of the methods’ predictions on external test sets.

The remainder of the paper is organized as follows: First, we interpret the methods’ results on the test and external sets and test significance in the observed differences. An in-depth discussion and the conclusions follow. Next, we give an overview of the used data sets and the design of the fair comparison framework. Towards the end of the article, we still give details of the included integration architectures.

## Results

### Architecture comparison

At first, we analyzed the results on the five cross-validation test sets (Tables 1, 2). Super.FELT and Omics Stacking achieved for two drugs the highest AUROCs, but for four drugs Super.FELT was the second best and Omics Stacking for one drug. MOLI, OmiEmbed and MOMA each achieved for one drug the highest AUROC, and EI and PCA not once. EI (mean rank = 5.71) and PCA (mean rank = 6.14) perform worse than the other methods. PCA reduces the view dimensions, but without taking into account the outcome, which can lead to poor results. The high-dimensional and sparse space of EI reduced its predictive performance.

OmiEmbed ranks in the midpoint, showing the difficulty of optimizing its *variational autoencoder* (VAE).

No method clearly outperforms the others, but Omics Stacking and Super.FELT performed slightly better, according to their mean ranks of 2.86 and 2.43, respectively.

The AUPRC results are similar, but the three algorithms that use triplet loss achieved the best result for five out seven drugs and EI was the the worst performing algorithm. The positive trend for methods using triplet loss shows its regularization benefit on discriminating responders and non-responders.

All architectures have a high standard deviation for AUROC and AUPRC, which underlines the importance of stratified cross-validation to alleviate the influence of data splitting.

**Table 1:** Mean AUROC on the test sets from cross-validation

Drug	Omics stacking	MOLI	Super.FELT	Early integration	OmiEmbed	MOMA	PCA
Gemcitabine TCGA	*0.646 ± 0.045*	0.628 ± 0.118	0.588 ± 0.070	0.611 ± 0.029	0.628 ± 0.057	**0.650 ± 0.029**	0.614 ± 0.075
Gemcitabine PDX	**0.651 ± 0.071**	0.622 ± 0.098	*0.646 ± 0.063*	0.586 ± 0.092	0.539 ± 0.078	0.625 ± 0.034	0.607 ± 0.057
Cisplatin	0.722 ± 0.066	**0.764 ± 0.039**	*0.753 ± 0.047*	0.660 ± 0.105	0.640 ± 0.071	0.714 ± 0.075	0.710 ± 0.072
Docetaxel	0.772 ± 0.077	0.792 ± 0.097	**0.813 ± 0.051**	0.731 ± 0.080	*0.803 ± 0.035*	0.783 ± 0.060	0.723 ± 0.043
Erlotinib	**0.754 ± 0.114**	0.705 ± 0.062	*0.744 ± 0.125*	0.671 ± 0.052	0.664 ± 0.130	0.739 ± 0.105	0.660 ± 0.069
Cetuximab	0.731 ± 0.090	0.731 ± 0.033	**0.768 ± 0.045**	0.677 ± 0.075	*0.754 ± 0.044*	0.751 ± 0.043	0.655 ± 0.062
Paclitaxel	0.667 ± 0.138	0.596 ± 0.117	*0.726 ± 0.121*	0.607 ± 0.060	**0.740 ± 0.098**	0.692 ± 0.081	0.588 ± 0.124
Mean Rank	*2.86*	3.86	**2**.**43**	5.71	4.00	3.00	6.14

Best results are shown in bold and second best are italics. The values represent the means and standard deviations over five iterations

**Table 2:** Mean AUPRC on the test sets from cross-validation

Drug	Omics stacking	MOLI	Super.FELT	Early integration	OmiEmbed	MOMA	PCA
Gemcitabine TCGA	*0.161 ± 0.053*	0.155 ± 0.065	0.108 ± 0.024	0.117 ± 0.025	0.132 ± 0.072	0.138 ± 0.041	**0.166 ± 0.084**
Gemcitabine PDX	*0.151 ± 0.085*	**0.154 ± 0.066**	0.130 ± 0.050	0.138 ± 0.046	0.082 ± 0.024	0.111 ± 0.027	0.141 ± 0.054
Cisplatin	*0.293 ± 0.089*	**0.316 ± 0.084**	0.282 ± 0.052	0.222 ± 0.065	0.204 ± 0.086	0.262 ± 0.075	0.710 ± 0.072
Docetaxel	0.316 ± 0.083	*0.345 ± 0.093*	**0.373 ± 0.027**	0.312 ± 0.117	0.251 ± 0.071	0.281 ± 0.090	0.278 ± 0.040
Erlotinib	*0.479 ± 0.153*	0.446 ± 0.108	**0.499 ± 0.162**	0.346 ± 0.120	0.468 ± 0.162	0.476 ± 0.151	0.347 ± 0.066
Cetuximab	*0.376 ± 0.104*	0.357 ± 0.075	**0.400 ± 0.095**	0.290 ± 0.057	0.329 ± 0.091	0.347 ± 0.070	0.259 ± 0.064
Paclitaxel	0.220 ± 0.108	0.163 ± 0.087	*0.245 ± 0.116*	0.160 ± 0.045	**0.270 ± 0.079**	0.213 ± 0.074	0.118 ± 0.049
Mean Rank	**2**.**29**	*2.86*	*2.86*	5.57	5.14	4.29	5.00

Best results are shown in bold and second best are italic. The values represent the means and standard deviations over five iterations

**Fig. 1:**
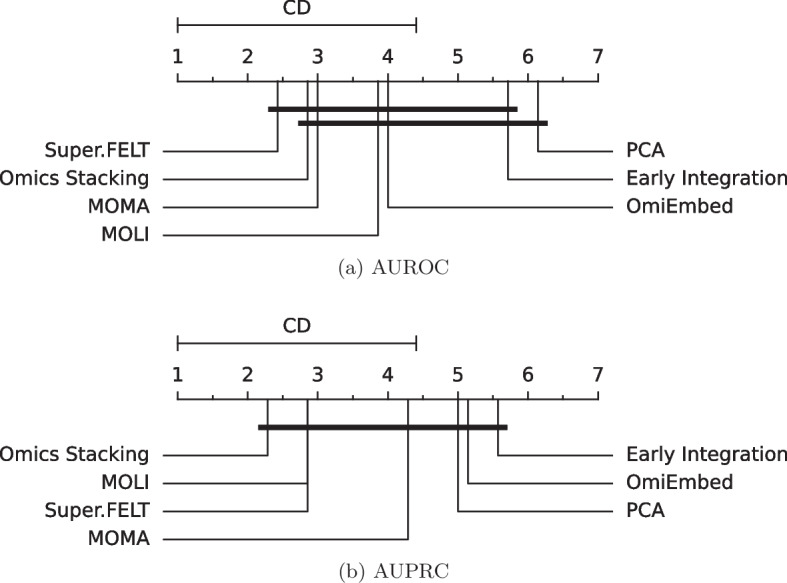
Mean rank and critical difference of the AUROC and AUPRC on the test sets from cross-validation. The mean values of the outer cross-validation results are compared. The Nemenyi test with $\alpha =0.05$ was used to compute significant differences

The visualization of the mean ranks and the critical differences (Fig. 1) supports our analysis. EI is, for AUROC as well as AUPRC, significantly worse than the best performing method. The mean ranks for AUPRC contain a visible gap between methods that use triplet loss (Omics Stacking, Super.FELT and MOLI) and methods without (MOMA, OmiEmbed and EI), but without significance.

Next, we analyzed the results on the external test sets (Tables 3, 4). Here, the method is trained to predict the *in vitro* response, but at the same time, has to learn general features that can predict drug response on an unknown *in vivo* distribution.

**Table 3:** Mean AUROC on external test set

Drug	Omics stacking	MOLI	Super.FELT	Early integration	OmiEmbed	MOMA	PCA
Gemcitabine TCGA	**0.655 ± 0.029**	*0.640 ± 0.037*	0.618 ± 0.042	0.604 ± 0.093	0.565 ± 0.059	0.473 ± 0.039	0.527 ± 0.085
Gemcitabine PDX	**0.714 ± 0.089**	0.614 ± 0.044	*0.692 ± 0.054*	0.525 ± 0.099	0.657 ± 0.119	0.627 ± 0.092	0.600 ± 0.088
Cisplatin	0.644 ± 0.087	0.674 ± 0.032	*0.728 ± 0.045*	0.604 ± 0.052	0.513 ± 0.056	0.687 ± 0.019	**0.743 ± 0.056**
Docetaxel	0.584 ± 0.101	**0.647 ± 0.038**	*0.588 ± 0.056*	0.456 ± 0.065	0.478 ± 0.056	0.581 ± 0.064	0.475 ± 0.122
Erlotinib	0.744 ± 0.065	0.722 ± 0.127	0.563 ± 0.080	*0.789 ± 0.079*	0.633 ± 0.105	0.715 ± 0.132	**0.800 ± 0.078**
Cetuximab	**0.575 ± 0.049**	0.476 ± 0.111	*0.556 ± 0.099*	0.470 ± 0.130	0.468 ± 0.075	0.505 ± 0.019	0.523 ± 0.134
Paclitaxel	**0.619 ± 0.152**	0.547 ± 0.121	0.527 ± 0.114	0.418 ± 0.062	0.516 ± 0.026	*0.573 ± 0.124*	0.435 ± 0.104
Mean Rank	**2**.**14**	3.43	*3.14*	5.57	5.43	4.14	4.14

Best results are shown in bold and second best are italic. The values represent the means and standard deviations over five iterations

**Table 4:** Mean AUPRC on external test set

Drug	Omics stacking	MOLI	Super.FELT	Early integration	OmiEmbed	MOMA	PCA
Gemcitabine TCGA	**0.581 ± 0.074**	*0.535 ± 0.085*	0.502 ± 0.082	0.513 ± 0.086	0.462 ± 0.095	0.389 ± 0.040	0.421 ± 0.081
Gemcitabine PDX	**0.510 ± 0.132**	0.424 ± 0.038	0.457 ± 0.055	0.362 ± 0.108	0.466 ± 0.100	0.414 ± 0.068	*0.481 ± 0.131*
Cisplatin	0.942 ± 0.027	0.950 ± 0.007	*0.963 ± 0.009*	0.932 ± 0.004	0.908 ± 0.009	0.952 ± 0.006	**0.964 ± 0.012**
Docetaxel	0.560 ± 0.051	**0.590 ± 0.021**	0.565 ± 0.024	0.491 ± 0.034	0.544 ± 0.049	*0.578 ± 0.060*	0.523 ± 0.089
Erlotinib	**0.440 ± 0.075**	0.410 ± 0.158	0.223 ± 0.024	*0.428 ± 0.106*	0.294 ± 0.079	0.369 ± 0.146	0.427 ± 0.138
Cetuximab	0.125 ± 0.018	*0.141 ± 0.086*	0.126 ± 0.039	0.108 ± 0.028	0.101 ± 0.018	**0.148 ± 0.065**	0.110 ± 0.032
Paclitaxel	**0.256 ± 0.147**	*0.191 ± 0.077*	0.147 ± 0.028	0.120 ± 0.016	0.135 ± 0.007	0.172 ± 0.047	0.163 ± 0.094
Mean Rank	**2**.**43**	*2.86*	4.00	5.43	5.57	3.86	3.86

Best results are shown in bold and second best are italic. The values represent the means and standard deviations over five iterations

**Fig. 2:**
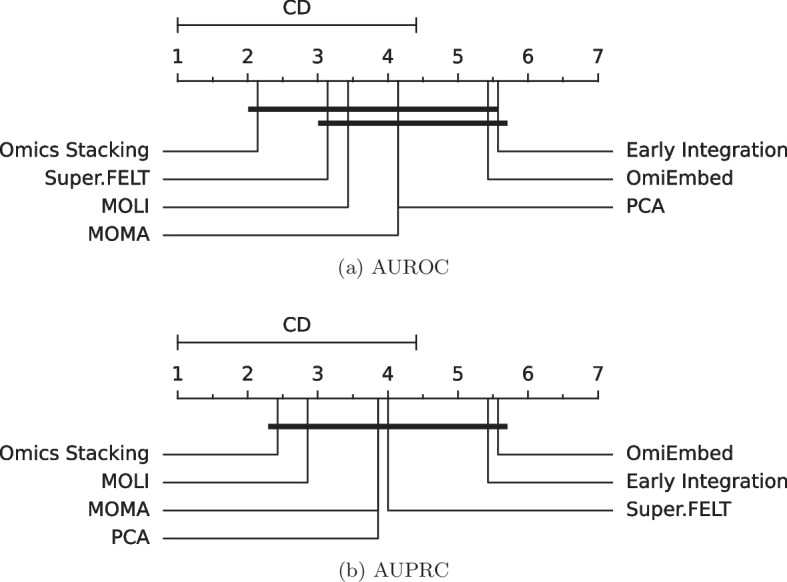
Mean rank and critical difference of the AUROC and AUPRC on the external test set. The mean values of the outer cross-validation results are compared. The Nemenyi test with $\alpha =0.05$ was used to compute significant differences

Again, no single method performed best with all drugs, but Omics Stacking had the lowest mean rank in both metrics, achieving the best results in more than half of the data sets for AUROC and AUPRC. Additionally, it achieved the lowest mean rank (see Fig. 2 for the critical differences) for AUROC and AUPRC. The results confirm the benefit of classifying both individual and integrated features for the translatability.

EI achieved the second best AUPRC and AUROC for Erlotinib, but classified worse in general. Surprisingly, OmiEmbed performed worst on the external data. One possible explanation may be that the regularization of the VAE narrows its capabilities to perform similarly on a shifted distribution without retraining.

PCA’s performance was better on the external data set than on the test set, where it was the worst method. We argue that PCA method did not overfit strongly on the train data, which increases its performance on the external data set and even achieved the best results for two drugs, but not enough to achieve acceptable predictions on all data sets.

Differences occurred between AUROC and AUPRC: Super.FELT had the second best mean rank for AUROC, but was only fourth for AUPRC. No method was significantly better regarding the AUPRC, however, OmiEmbed and EI had similarly low mean ranks. Omics Stacking performed best for the AUPRC and AUROC.

### Omics stacking ablation study

We performed an ablation study to test the impact of different components of Omics Stacking, our newly developed integration architecture. We wanted to measure in particular the influence of different numbers of stacked classification layers and the triplet loss on the predictive performance. The hyperparameter optimization framework and data sets were the same as in the architecture comparison.

The first altered architecture—Omics Stacking without Integration—omits the subnetwork that classifies the concatenated omics features, which results in a late integration neural network. The second one—Omics Stacking Complete—adds additional classifiers for the integration of two omics, which adds three classification subnetworks: expression and mutation, expression and CNA, and mutation and CNA. At last, we also tested Omics Stacking without triplet loss.

**Fig. 3:**
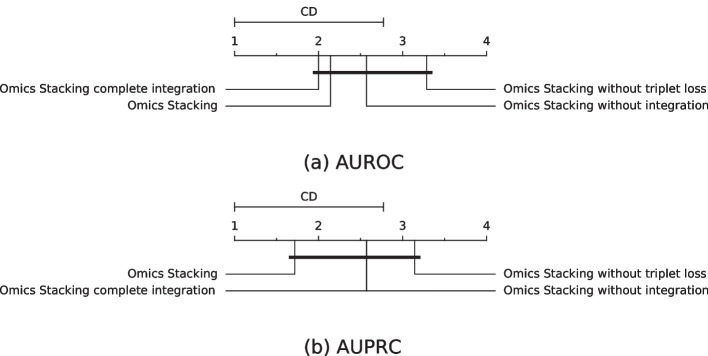
Mean rank and critical difference of the AUROC and AUPRC on the test sets from cross-validation for the ablation study. The mean values of the outer cross-validation results are compared. The Nemenyi test with $\alpha =0.05$ was used to compute significant differences

**Fig. 4:**
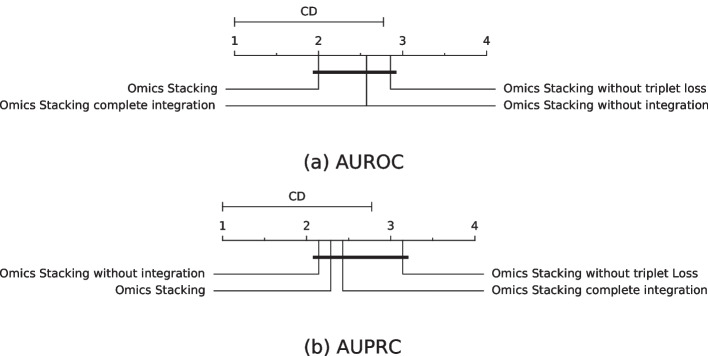
Mean rank and critical difference of the AUROC and AUPRC on the external test data for the ablation study. The mean values of the outer cross-validation results are compared. The Nemenyi test with $\alpha =0.05$ was used to compute significant differences

The AUROCs, AUPRCs and mean ranks are given in Tables 5, 6, 7 and 8 and the visualization of them in Figs. 3 and 4.

Both Omics Stacking Complete Integration and Omics Stacking without Integration achieved the best rank once and Omics Stacking without triplet Loss always had the lowest mean rank. Omics Stacking achieved twice the first place in the ranking and was never worse than second place.

In the end, none of the different configurations achieved a significantly better or worse performance, but the trend indicates that triplet loss and multi-omics integration increases the prediction, because removing them always decreased the mean ranking.

**Table 5:** Mean AUROC on test sets from cross-validation for the ablation study

Drug	Omics stacking			
	Complete integration	Without integration	Integration	Without triplet loss
Gemcitabine TCGA	*0.630 ± 0.055*	0.605 ± 0.076	**0.646 ± 0.045**	0.601 ± 0.084
Gemcitabine PDX	0.640 ± 0.089	**0.656 ± 0.062**	*0.651 ± 0.071*	0.593 ± 0.044
Cisplatin	*0.742 ± 0.050*	**0.757 ± 0.061**	0.722 ± 0.066	0.734 ± 0.091
Docetaxel	*0.775 ± 0.089*	0.759 ± 0.031	0.772 ± 0.077	**0.813 ± 0.024**
Erlotinib	0.696 ± 0.101	*0.744 ± 0.098*	**0.754 ± 0.114**	0.662 ± 0.112
Cetuximab	**0.748 ± 0.048**	0.679 ± 0.052	0.731 ± 0.090	0.721 ± 0.055
Paclitaxel	**0.695 ± 0.104**	0.634 ± 0.114	*0.667 ± 0.138*	0.522 ± 0.085
Rank	**2**.**00**	2.57	*2.14*	3.29

Best results are shown in bold and second best are italic. The values represent the means and standard deviations over five iterations

**Table 6:** Mean AUPRC on test sets from cross-validation for the ablation study

Drug	Omics stacking			
	Complete integration	Without integration	Integration	Without triplet loss
Gemcitabine TCGA	*0.143 ± 0.053*	0.136 ± 0.054	**0.161 ± 0.053**	0.149 ± 0.074
Gemcitabine PDX	0.138 ± 0.046	**0.204 ± 0.092**	*0.151 ± 0.085*	0.119 ± 0.046
Cisplatin	0.288 ± 0.072	**0.293 ± 0.074**	**0.293 ± 0.089**	0.277 ± 0.084
Docetaxel	*0.317 ± 0.064*	0.262 ± 0.072	0.316 ± 0.083	**0.358 ± 0.046**
Erlotinib	0.422 ± 0.704	*0.440 ± 0.149*	**0.479 ± 0.153**	0.352 ± 0.111
Cetuximab	**0.379 ± 0.102**	0.264 ± 0.042	*0.376 ± 0.104*	0.350 ± 0.049
Paclitaxel	0.186 ± 0.076	*0.188 ± 0.103*	**0.220 ± 0.108**	0.090 ± 0.024
Rank	*2.57*	*2.57*	**1**.**71**	3.14

Best results are shown in bold and second best are italic. The values represent the means and standard deviations over five iterations

**Table 7:** Mean AUROC on external test set for the ablation study

Drug	Omics stacking			
	Complete integration	Without integration	Integration	Without triplet loss
Gemcitabine TCGA	*0.646 ± 0.048*	0.641 ± 0.056	**0.655 ± 0.029**	0.624 ± 0.068
Gemcitabine PDX	0.656 ± 0.049	0.630 ± 0.066	**0.714 ± 0.089**	*0.665 ± 0.078*
Cisplatin	*0.668 ± 0.046*	**0.685 ± 0.078**	0.644 ± 0.087	0.680 ± 0.063
Docetaxel	**0.613 ± 0.058**	0.597 ± 0.046	0.584 ± 0.101	*0.600 ± 0.090*
Erlotinib	0.704 ± 0.141	0.715 ± 0.193	**0.744 ± 0.065**	*0.741 ± 0.077*
Cetuximab	0.529 ± 0.052	**0.599 ± 0.173**	*0.575 ± 0.049*	0.466 ± 0.122
Paclitaxel	*0.511 ± 0.106*	0.432 ± 0.087	**0.619 ± 0.152**	0.419 ± 0.082
Rank	*2.57*	*2.57*	**2**.**00**	2.86

Best results are shown in bold and second best are italic. The values represent the means and standard deviations over five iterations

**Table 8:** Mean AUPRC on the test sets from cross-validation for the ablation study

Drug	Omics stacking			
	Complete integration	Without integration	Integration	Without triplet loss
Gemcitabine TCGA	0.537 ± 0.040	*0.545 ± 0.088*	**0.581 ± 0.074**	0.489 ± 0.070
Gemcitabine PDX	**0.522 ± 0.087**	0.455 ± 0.072	*0.510 ± 0.132*	0.483 ± 0.109
Cisplatin	*0.954 ± 0.009*	0.953 ± 0.022	0.942 ± 0.027	**0.955 ± 0.012**
Docetaxel	*0.571 ± 0.028*	**0.644 ± 0.048**	0.560 ± 0.051	0.568 ± 0.056
Erlotinib	0.400 ± 0.171	**0.482 ± 0.251**	*0.440 ± 0.075*	0.421 ± 0.126
Cetuximab	0.107 ± 0.012	**0.188 ± 0.164**	*0.125 ± 0.018*	0.102 ± 0.024
Paclitaxel	*0.176 ± 0.094*	0.127 ± 0.027	**0.256 ± 0.147**	0.121 ± 0.017
Rank	2.43	**2**.**14**	*2.29*	3.14

Best results are shown in bold and second best are italic. The values represent the means and standard deviations over five iterations

### Triplet loss ablation study

The architecture comparison indicated a trend favoring architectures that were trained with triplet loss. We performed an ablation study in which we evaluated the impact of triplet loss separately, for each architecture. For the study we removed the triplet loss from architectures that already used it and added it to the others.

Removing the triplet loss from MOLI and Omics Stacking is easily done by omitting the term from the loss computation. To remove the triplet loss from Super.FELT, we exchanged the supervised encoder of the first phase with unsupervised autoencoders.

For MOMA, we computed the triplet loss on the concatenated vectors before the attention mechanism and for OmiEmbed, on the concatenated vectors before the computation of $\mu$ and $\sigma$. We left out PCA, as the principal components need not be computed by gradient descent.

We calculated the paired samples differences and counted the number of times the difference was positive, which means that triplet loss improved the result, to see whether there exists a general trend. Additionally, we performed a two-sided Wilcoxon signed-rank test, with $\alpha <0.05$ for significance, on the paired results per architecture (Table 9). The Wilcoxon signed-rank test includes the differences of the paired samples, which has the advantage that it also incorporates the improvement or degradation of the test metric.

Additional file 1 presents the detailed results of the adapted architectures.

**Table 9:** Comparison of *p* values for all architectures computing the triplet loss or not

Data	Method	AUROC	AUPRC
Test	Early integration	**0**.**047** (6/7)	**0**.**016** (7/7)
	Omics stacking	0.156 (5/7)	0.156 (6/7)
	Super.FELT	0.219 (5/7)	0.813 (4/7)
	MOLI	0.813 (4/7)	0.813 (3/7)
	OmiEmbed	0.938 (3/7)	0.578 (4/7)
	MOMA	0.296 (2/7)	0.078 (1/7)
External	Omics stacking	0.219 (5/7)	0.078 (5/7)
	MOLI	0.463 (4/7)	0.297 (6/7)
	Super.FELT	0.578 (4/7)	0.938 (4/7)
	OmiEmbed	0.578 (4/7)	0.938 (4/7)
	MOMA	0.375 (2/7)	0.578 (2/7)
	Early integration	0.156 (1/7)	0.219 (1/7)

The *p* values are from a Wilcoxon signed-rank test, where an $\alpha <0.05$ was regarded as significant. Additionally, we counted the number of times the method was better using the triplet loss and added it in parentheses. The methods are sorted in descending order with the criterion of the times the triplet loss improved the results separately for test and external data

In the case of EI, adding the triplet loss improves the results with regard to the AUROC in six out of seven cases, and for the AUPRC, in all cases. For the test data, the improvements were statistically significant across the board. For external data, the trend is reversed and no statistically significant different can be observed. The regularization helped EI to distinguish elements of the test data, but did not learn generalizable features, which reduced its capabilities to predict the response on external data.

Omics Stacking profited in both cases from the triplet loss, but not with significant differences. For MOLI and Super.FELT, the results were in half or slightly more than half of the cases improved. The results for MOMA and OmiEmbed were the other way around: In half or less than half of the cases they improved with triplet loss. Both methods already have a composed loss function and adding another term increases the difficulty improving upon it.

### Interpretability

For all of the above models and architectures, it would be desirable to compute the importance and contribution of individual variables to the outcome of a prediction model. This problem is addressed in the field of explainable AI (XAI) by so-called *attribution methods*. Attribution methods for architectures computing and concatenating latent representations from thousands of variables are, however, still in their infancy [12]. Therefore, we instead focus on the *attribution of views (omics), not individual features*. In the following, we analyze the sum of attributions as well as the mean attribution of features from various views.

To train the final model we computed the final hyperparameter set for an architecture with a 5-fold cross-validation and the same hyperparameter optimization. The final model was trained on the complete GDSC data and the Shapley values [13] were computed on the external data.

Shapley values are a concept from cooperative game theory and can be used to interpret the predictions of a machine learning model. The Shapley value of a feature represents its attribution to the prediction. Shapley values are usually computed in a model-agnostic manner, by changing the inputs and measuring the changes in the models’ output.

For the Shapley value combination, we employ a perturbation based approach, where features are added one by one to a baseline. The order in which features are added is changed randomly in every iteration, and the mean of all attributions represents the final attribution of a feature [13]. In the following, we refer to this method as *Shapley sampling*.

The Shapley values of an architecture for one drug were normalized so that they sum up to one. We took the sum of an omics’ attributions to analyze its influence on the drug response prediction and the mean of an omics’ attributions to evaluate the average influence of its features on the result. Subsequently, the mean of both values over all drug data sets were calculated.

It is important to notice that after the variance threshold, the number of features for mutation (10–15 thousand) and CNA (18–22 thousand) were one magnitude higher than for expression (around three thousand). Additional files 2, 3, 4, 5, 6, 7 and 8 list the gene level attributions per drug for all architectures.

For EI, Shapley sampling assigns the highest attribution to the CNA view (0.642), and less to the expression (0.155) and the mutation view (0.203). However, the mean influence of an expression feature is greater than the mean influence of a copy number or mutation feature (0.00005 vs. 0.00003 resp. 0.00001). This indicates that EI learns that the gene expression is an important indicator for the drug response [9, 14], but in general the other views have a higher influence.

MOMA focuses mostly on two omics (mutation = 0.442, expression = 0.462), due its attention mechanism, and less on CNA (0.094). PCA is the only architecture that uses expression (0.496) and CNA (0.467) equally.

The attributions for MOLI, Super.FELT and OmiEmbed show similar tendencies. The sum of the attributions of expression makes up more than half of the whole attribution. The mean attribution of an expression feature is up to approximately twenty times higher than that of a copy number or mutation feature. The intermediate integration does not favor views with many features and learns which inputs are not relevant for the drug response prediction.

Omics Stacking distributes the attribution more evenly on expression (0.518), mutation (0.210) and CNA (0.272). Its summarized attribution has a high variance, indicating that the meta learner learns individually for each data set to focus on the most important omics. Similar to the other intermediate integration architectures, the averaged attributions for expression features are considerably higher than the others.

The diagrams in Additional file 9 visualize the summarized and averaged attributions.

## Discussion

The emerging interest in multi-omics integration with neural networks produces an increasing number of different architectures. In this work, we compared a subset of recently published methods as fairly as possible to validate their predictive capabilities. Our experiments focused on drug response prediction, a high-dimensional problem, with the added complexity of having few samples.

The performance was only tested on data sets from cancer research mainly due to two reasons: First, the availability of multi-omics data, as cancer samples are more common compared to other diseases and data sets for other diseases with enough samples and a high enough quality are still rare. The second reason is that the extensive hyperparameter optimization with an inner and outer cross-validation increases the hardware requirements greatly and combined with the sheer amount of different, recently published algorithms made it necessary to focus on just a single field.

We focused our research on the comparison of the predictive performance and the summarized attributions, but another important aspect is to analyze the attribution on a more fine-granular level, i.e., on the level of genes. As this is a research topic of its own, especially in the context of high-dimensional data and autoencoder-type architectures, we have to leave that for future work.

Current drug response methods are trained on cell lines only and applied directly to patient samples without adaptation. A different strategy is transfer learning, where information from source data is transferred to target data for improved prediction [15]. This can be useful for transferring information from *in vitro* to *in vivo* samples. However, larger *in vivo* or *ex vivo* data sets would be necessary for this to succeed.

Our experiments validate results that using EI results in worse predictions than intermediate integration, because concatenating the views creates a higher-dimensional sparse feature space that further increases the issue of having only few samples. EI is suitable to be used as simple and fast to implement baseline, but at least one intermediate integration architecture should be validated. However, making a recommendation for one of them is difficult, as none of them performed significantly better.

## Conclusions

In this paper we showed that none of the current multi-omics integration methods excels at drug response prediction, however, Early Integration and PCA performed significantly worse.

Researchers should not rely on a single method, but rather consider more than one method for their task at hand. If the number of experiments is limited or translatability is wanted, we recommend to use the newly introduced method, Omics Stacking, as it achieved good results on test and external data. When faced with a new data set in a cross-validation like setting, Super.FELT is another good option.

Our experiments also suggest that a fair experimental is necessary to see the strengths and weaknesses of various algorithms, which were not visible from the publications alone. We hope that this comparison has shed some light on the relative performance of multi-omics integration methods, has produced valuable insights for their application, and that it encourages further research.

## Methods

### Drug response data

For our experiments, we used a publicly available drug response data set containing responses for six drugs: Docetaxel, Cisplatin, Gemcitabine, Paclitaxel, Erlotinib, and Cetuximab [16]. The data set was chosen, because it had *patient-derived xenograft* (PDX) or patient data necessary to test the method’s translatability [4]. Two external test sets are available for Gemcitabine.

The data set contains the drug response as target and data for three omics: somatic point mutations, somatic copy number profiles and gene expression profiles. Gene expressions are standardized and pairwise homogenized. Gene-level copy number estimates are binarized, representing copy-neutral genes as zeroes and genes with overlapping deletions or amplifications as ones. Somatic point mutations are also in a binary format: one for mutated genes and zero for genes without a mutation [4]. For a fair comparison, the same data processing and training procedures were used for all methods. We used a variance threshold to filter genes with a small variance, as they hardly provide additional information. We set the variance thresholds in the same way as Park et al.  [17]. After the variance threshold around three thousand features for gene expression remained, around 14–18 thousand for mutation and around 15 up 22 thousand for CNA depending on the used data set.

Sharifi-Noghabi et al. acquired the data from PDX mice models [18], *The Cancer Genome Atlas* (TCGA) [19] and *Genomics of Drug Sensitivity in Cancer* (GDSC) [20]. GDSC consists of cell line data and was used for training, validation and testing because of its high number of samples. The trained neural networks were additionally tested on either PDX or TCGA to validate the algorithms’ translatability.

The characteristics of the data set per drug are summarized in Table 10.

**Table 10:** Characteristics of the drug response multi-omics data set

Drug	Resource	Number of Samples	Usage
Cetuximab	GDSC	856 (NR:735, R:121)	Train & test
Cisplatin	GDSC	829 (NR:752, R:77)	Train & test
Docetaxel	GDSC	829 (NR:764, R:65)	Train & test
Erlotinib	GDSC	362 (NR:298, R:64)	Train & test
Gemcitabine	GDSC	844 (NR:790, R:54)	Train & test
Paclitaxel	GDSC	389 (NR:363, R:26)	Train & test
Cetuximab	PDX	60 (NR:55, R:5)	External test
Cisplatin	TCGA	66 (NR:6, R:60)	External test
Docetaxel	TCGA	16 (NR:8, R:8)	External test
Erlotinib	PDX	21 (NR:18, R:3)	External test
Gemcitabine	PDX	25 (NR:18, R:7)	External test
Gemcitabine	TCGA	57 (NR:36, R:21)	External test
Paclitaxel	PDX	43 (NR:38, R:5)	External test

NR, non-responder; R, responder

### Comparison framework

To compare the algorithms fairly, different precautions were taken: First, the same preprocessing was performed in all experiments to provide the same input data, and second, the hyperparameters of the algorithms were optimized with an equal number of iterations by random search from a fixed grid. All algorithms draw parameters from the same grids (Table 11).

**Table 11:** The hyperparameter grid used in the hyperparameter optimization

Parameter	Values
Batch size	$\{8, 16, 32\}$
Dropout rate	$\{0.1, 0.3, 0.5, 0.7\}$
Epochs	$\{2..20\}$
Gamma	$\{0.0, 0.1, 0.3, 0.5\}$
Layer dimension	$\{32, 64, 128, 256, 512, 1024\}$
Learning rate	$\{0.001, 0.01\}$
Margin	$\{0.2, 0.5, 1\}$
Weight decay	$\{0.0001, 0.001, 0.01, 0.05, 0.1\}$
PCA variance threshold	$\{0.9, 0.95, 0.975, 0.99\}$

A $5\times 5$ stratified cross-validation was performed to reduce the dependence on the data splitting. 200 hyperparameter sets were created for each iteration of the outer cross-validation and each set was used for training in the inner cross-validation. The mean *area under the receiver operating characteristic* (AUROC) was used as performance measure. The algorithm was retrained with the best hyperparameter set on the combined train and validation sets. The trained network was used to compute the final results on the test sets from cross-validation and the external test set.

The data sets are imbalanced as they contain only few responders. In the drug discovery process, researchers are mainly interested in the positive samples to find an effective drug. To account for this requirement, the *area under precision recall curve* (AUPRC) [21] was additionally computed.

One requirement to use these methods is for them to work on patient data sets which are normally too small for transfer learning and still detect positive samples. This was covered by using the final model of an outer cross-validation iteration on an external patient or PDX data set.

The comparison of multiple algorithms on multiple data sets can lead to ambiguous results, if none of them performs significantly better. We compute the mean ranking as single value for a better comparison. Additionally, we compute the critical difference (CD) [8] with the Nemenyi significance test with $\alpha = 0.05$.

### Multi-omics integration architectures

Most deep learning algorithms for multi-omics integration are based upon the concept of encoding the feature space into a lower-dimensional latent space. The encoded representation of features in the latent space is commonly called latent features. The subnetwork that computes the latent features is called encoder and computes a non-linear dimensionality reduction. The smaller dimension of the latent representation assures that the encoder does not simply learn the identity function. An autoencoder transforms an input first into the latent representation and then decodes it back into the input dimension. The reconstructed sample should resemble the input as far as possible. The difference between input and reconstruction is measured and used as loss function [22].

Next, we explain briefly the seven multi-omics integration architectures we compared. The first one is *Early integration* (EI), which serves as baseline in our experiments. EI concatenates the omics before they serve as single input of a neural network, which consists of an encoding subnetwork and a classifier layer. The network is trained by minimizing the binary cross-entropy loss function, given that our target is to classify subjects into responders and non-responders.

**Fig. 5:**
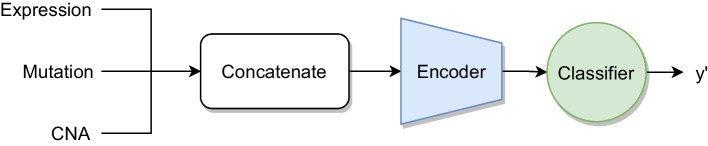
Schematic architecture of early integration with three input omics

The schematic architecture for three omics in visualized in Fig. 5.

**Fig. 6:**
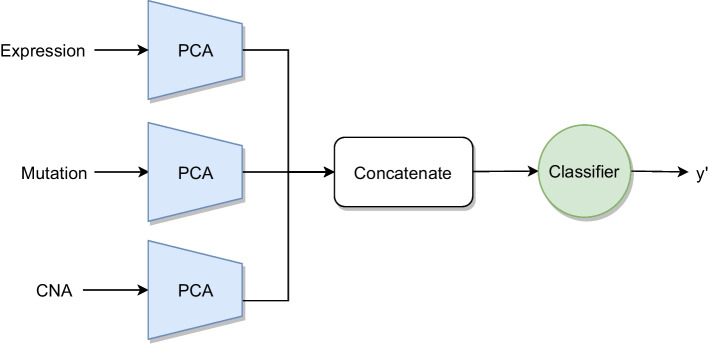
Schematic architecture of PCA integration with three input omics

An architecture based on *principal components analysis* (PCA) for intermediate integration was tested. After computing the PCA for each view individually, the resulting principal components are concatenated to obtain a feature representation. Subsequently, this feature representation is used to train a classifier. The scheme of PCA intermediate integration is visualized in Fig. 6.

**Fig. 7:**
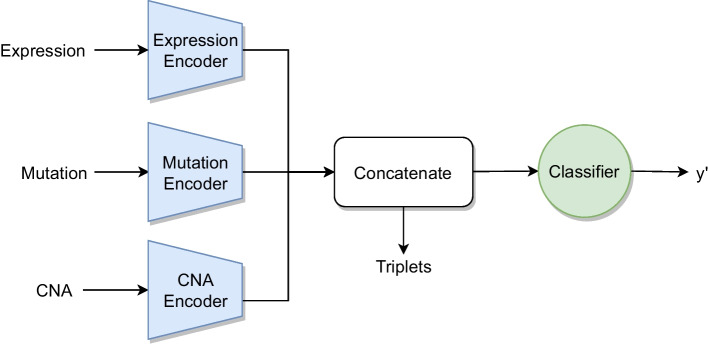
Schematic architecture of MOLI with three input omics

The next method is *Multi-Omics Late Integration* (MOLI) developed by Sharifi-Noghabi et al. [4], which is, notwithstanding its name, an intermediate integration method. MOLI uses an individual encoder for each omics to compute the latent representations, which are concatenated and used as input for the classifier (Fig. 7).

Due to the small sample size, the neural network is prone to overfitting, so MOLI uses the triplet loss [23] for regularization. The rationale behind triplet loss is that instances of the same class should have shorter distance between them than instances of different classes. To calculate the loss value, the triplet loss uses the embedding $f(x) \in {\mathbb {R}}^d$ of three samples. The first one is the anchor sample $x_i^a$, the second $x_i^p$ belongs to the same class, and at last a sample $x_i^n$ is of the opposite class.

**Fig. 8:**
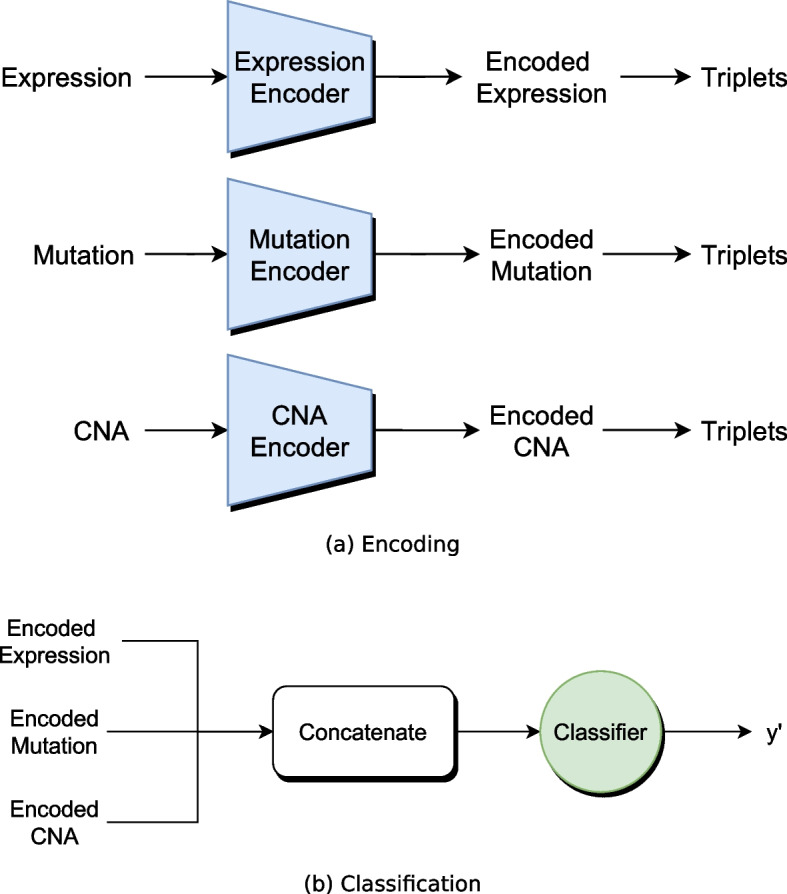
Schematic architecture of Super.FELT with three input omics

With them, the following loss function is minimized:

1 $$\begin{aligned} {\mathcal {L}}_{triplet}= \sum _i^N \left[ ||f(x_i^a)-f(x_i^p)||_2^2- ||f(x_i^a) - f(x_i^n)||_2^2 + \alpha \right] _+, \end{aligned}$$

where $\alpha$ is a margin that defines the minimum distance between pairs of different classes. In MOLI the concatenated latent representations are used as embeddings. The final loss function is the sum of classification loss and triplet loss:

2 $$\begin{aligned} {\mathcal {L}}_{MOLI} = {\mathcal {L}}_{Classification} + \gamma {\mathcal {L}}_{triplet}, \end{aligned}$$

where the influence of the triplet loss is weighted by the hyperparameter $\gamma$ and ${\mathcal {L}}_{Classification}$ is the binary cross-entropy. *Supervised Feature Extraction Learning using triplet loss* (Super.FELT) is a variation of MOLI, in which the encoding and classification is not performed jointly, but in two different phases [17]. The first phase trains a supervised encoder with triplet loss for each omics to extract latent features of the high-dimensional omics data to avoid overfitting. The latent features are concatenated and used to train a classifier. Figure 8a shows the encoding phase and (b) the classification phase of Super.FELT. A distinct neural network need to be trained for encoding an omics samples and another one for the classification of the integrated latent features, which increases in comparison to MOLI the training time.

Super.FELT added a variance threshold in the feature selection to remove features with a low variance [17]. It is based on the assumption that genes with a low variance might contain less important information and can be safely removed to reduce the dimensionality, hence increasing the focus on the more variable genes.

Additionally, we developed a novel extension of MOLI, which we call Omics Stacking. It is inspired by stacking and a combination of intermediate and late integration. The method stacks the results of different neural network classifier layers that use different latent features as input, with a meta-learner. The meta-learner can be any classifier, but we opted for a fully connected layer to train the neural network end-to-end.

**Fig. 9:**
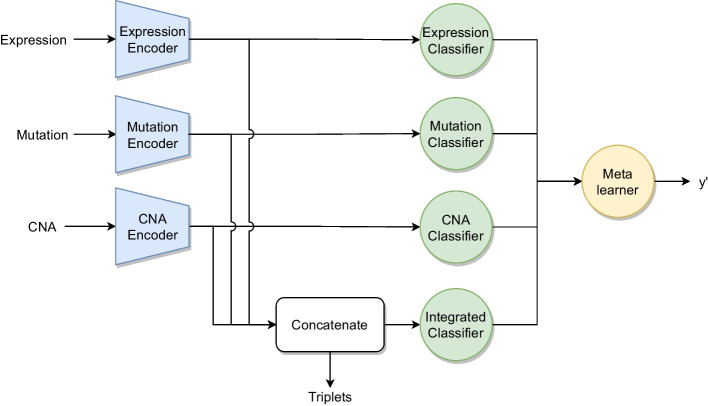
Schematic architecture of Omics-Stacking with three input omics

The omics are transformed to the latent space with individual encoders, but instead of only classifying the concatenated features, Omics Stacking trains a separate classifier for each omics and the concatenated omics (Fig. 9). The triplet loss is still used on the concatenated embeddings to regularize. The outputs of the classifiers are combined by a meta-learner that enables the weighting of different results to emphasize the most accurate one.

Omics Stacking combines the advantages of intermediate and late integration. It models the interaction between omics and retains the weak signals of individual omics, hence, capturing shared and complementary information. It relies not only on the combined omics, but also on the individual omics and so fosters generalization.

The former methods were developed and tested on drug response prediction, but in fact can be used for other tasks as well. Next we present two methods that were validated for the prediction of a tumor’s type or stage.

The first of these methods, *Multi-task Attention Learning Algorithm for Multi-omics Data* (MOMA) [24], uses a geometrical representation and an attention mechanism [25]. It is composed of three components: First, it builds a module for each omics using a module encoder. Each omics has its own module encoder consisting of two fully connected layers that convert omics features to modules. A module is represented by a normalized two-dimensional vector.

**Fig. 10:**
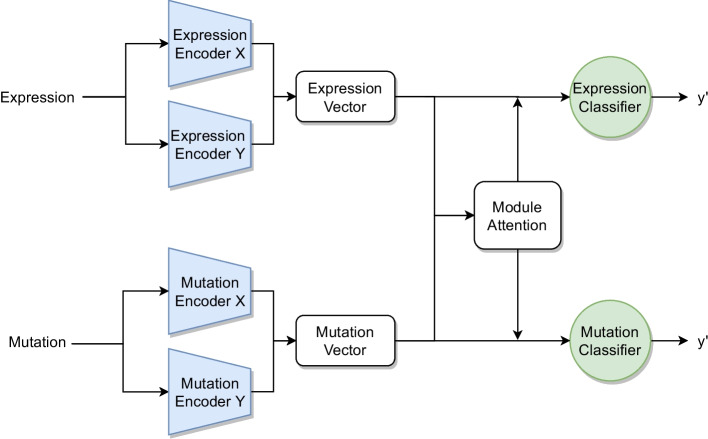
Schematic architecture of the network used in MOMA for two input omics

Second, it focuses on important modules between omics using a module attention mechanism. This mechanism is designed to act as a mediator to identify modules with high similarity among multiple omics. The relevance between modules is measured by the cosine similarity and is converted to a probability distribution with the softmax function. The distributions are then used to create an attention matrix that stores the relationship information between modules of different omics. To highlight important modules, the module vectors are multiplied by the attention matrix (Fig. 10).

Subsequently, fully connected layers and the logistic function are applied to flatten the multidimensional vectors and to compute the final probabilities for each omics [24].

MOMA is trained with cross-entropy loss between the true label and the omics specific outputs. After the training of the neural network, a logistic regression is fit on the omics specific outputs to generate the combined prediction.

**Fig. 11:**
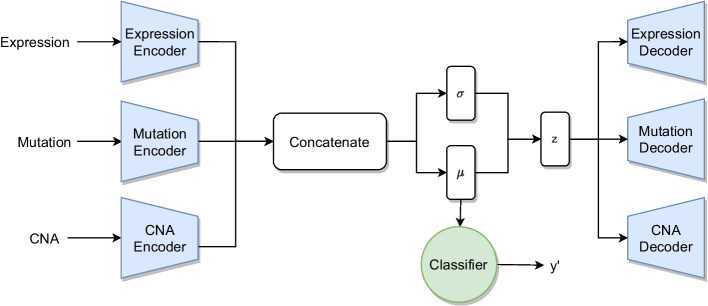
Schematic architecture of OmiEmbed for three input omics

The last architecture we tested OmiEmbed [26], which is based on a supervised variational autoencoder [27]. OmiEmbed was developed as a unified end-to-end multi-view multitask deep learning framework for high-dimensional multi-omics data. The method learns the latent features with the auxiliary unsupervised task of reconstructing the input omics. The trained latent features can be used for one or more supervised tasks. The overall architecture of OmiEmbed comprises a deep embedding module and one or multiple downstream task modules (Fig. 11).

The loss function of OmiEmbed is the sum of two parts: the reconstruction loss and the downstream task loss. ${\mathcal {L}}_{embed}$ is the unsupervised loss function of the VAE:

3 $$\begin{aligned} {\mathcal {L}}_{embed} = \frac{1}{M} \sum _{i=1}^{M} BCE(x_i, x_i') + D_{KL} ({\mathcal {N}}(\mu , \sigma )||{\mathcal {N}}(0,1)). \end{aligned}$$

*BCE* is the binary cross-entropy to measure the differences between the input *x* and the reconstruction $x'$ and is computed individually for each of the *M* omics. $D_{KL}$ is the Kullback–Leibler divergence between the learned distribution and a standard normal distribution.

The embedding loss function is used together with the loss of the downstream task, which is in our case classification:

4 $$\begin{aligned} {\mathcal {L}}_{total} = \lambda {\mathcal {L}}_{embed} + {\mathcal {L}}_{CE}, \end{aligned}$$

where ${\mathcal {L}}_{CE}$ is the cross-entropy loss and $\lambda$ a balancing weight.

Instead of training all layers at the same time, the network learns in three phases: First, only the VAE is trained. In the second phase, the VAE weights are fixed and only the downstream network is trained and in the last phase the complete network is fine-tuned.

Table 12 summarizes the components and architectures of the described methods.

**Table 12:** Characteristics of the multi-omics integration methods

Architecture	Training	Triplet loss	Integration type	Encoding
Early integration	End-to-end	–	Early	Supervised encoder
MOLI	End-to-end	+	Intermediate	Supervised encoder
Super.FELT	Encoding and classifying	+	Intermediate	Supervised encoder
Omics stacking	End-to-end	+	Intermediate + late	Supervised encoder
MOMA	End-to-end	–	Intermediate + late	Vector encoding
OmiEmbed	Three phases	–	Intermediate	Variational supervised autoencoder
PCA	PCA and classifier	–	Intermediate	Principal components

### Implementation details

The algorithms were implemented in PyTorch 1.11.0 and NumPy 1.22.4 was used to compute the AUROC and AUPRC. The critical differences were computed and visualized with Orange3 3.32.0. The Shapley values were computed with Shapley value sampling with 50 permutations of Captum 0.5.0.

The triplets were generated online with an all-triplets scheme, which creates all possible triplets of a batch, as in the experiments of the corresponding paper of MOLI and Super.FELT. An open source implementation of triplet loss and triplet creation was used.^1^ Adagrad [28] was used to update the neural network weights.

Early Integration and Omics Stacking were implemented by the authors. For MOMA we used the provided example source code^2^ and adapted it to three omics, made the number of modules a hyperparameter and combined the individual probability outputs with a logistic regression. For OmiEmbed,^3^ MOLI^4^ and Super.FELT,^5^ the published source code was used.

To reduce the optimization runtime, we applied an early stopping scheme in the inner cross-validation. If it was not possible anymore to improve on the currently best mean AUROC for a hyperparameter set – even with best results (an AUROC of 1) on all remaining sets – the inner cross-validation terminates early and the optimization continues with the next hyperparameter set.

## Supplementary Information

**Additional file 1.** Triplet loss ablation study: AUROC and AUPRC values for all architectures with and without triplet loss.

**Additional file 2.** Attributions for Cetuximab: Gene level attributions defined by Shapley values.

**Additional file 3.** Attributions for Cisplatin: Gene level attributions defined by Shapley values.

**Additional file 4.** Attributions for Docetaxel: Gene level attributions defined by Shapley values.

**Additional file 5.** Attributions for Erlotinib: Gene level attributions defined by Shapley values.

**Additional file 6.** Attributions for Gemcitabine PDX: Gene level attributions defined by Shapley values.

**Additional file 7.** Attributions for Gemcitabine TCGA: Gene level attributions defined by Shapley values.

**Additional file 8.** Attributions for Paclitaxel: Gene level attributions defined by Shapley values.

**Additional file 9.** Visualization of the aggregated attributions: Mean and standard deviation over all drug data sets of each omics mean and summarized attributions.

## Data Availability

The datasets analyzed during the current study are available on Zenodo, https://doi.org/10.5281/zenodo.4036592. The raw data is based on: GDSC (https://www.cancerrxgene.org/), the Supplementary of Gao et al. [18] (https://www.nature.com/articles/nm.3954), ArrayExpress (https://www.ebi.ac.uk/arrayexpress/) and Firehose Broad GDAC (http://gdac.broadinstitute.org/runs/stddata__2016_01_28/data/).

## Footnotes

1 https://github.com/adambielski/siamese-triplet.

2 https://github.com/DMCB-GIST/MOMA.

3 https://github.com/zhangxiaoyu11/OmiEmbed.

4 https://github.com/hosseinshn/MOLI.

5 https://github.com/DMCB-GIST/Super.FELT.
