# Supplementary material for: A fair experimental comparison of neural network architectures for latent representations of multi-omics for drug response prediction
Source: BMC Bioinformatics. 2023 Feb 14;24:45. doi: 10.1186/s12859-023-05166-7 (PMC9926634; doi:10.1186/s12859-023-05166-7)
Supplement: Supplementary file 9 — Additional file 9. Visualization of the aggregated attributions: Mean and standard deviation over all drug data sets of each omics mean and summarized attributions. [file 12859_2023_5166_MOESM9_ESM.pdf]

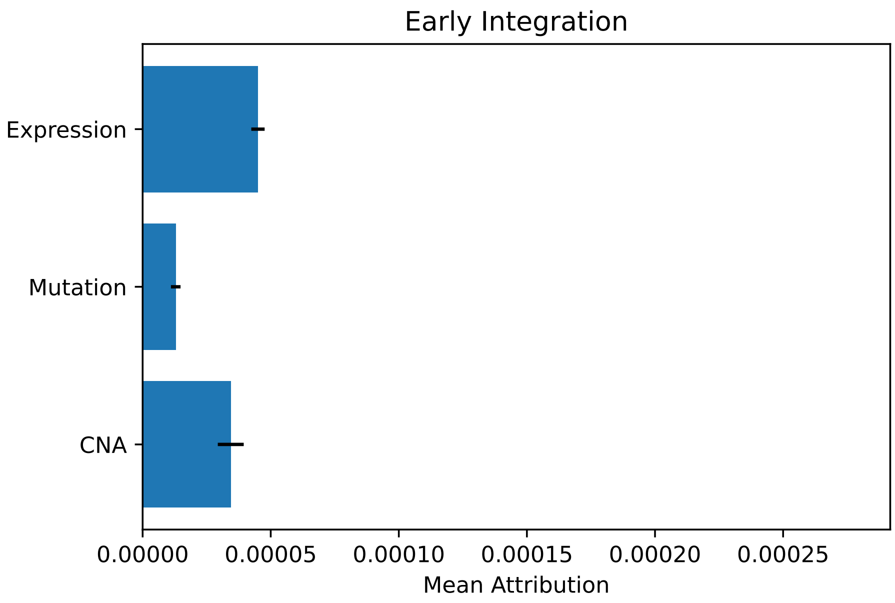

Figure 1: Mean and standard deviation over all drug data sets of each omics' mean attributions for Early Integration.

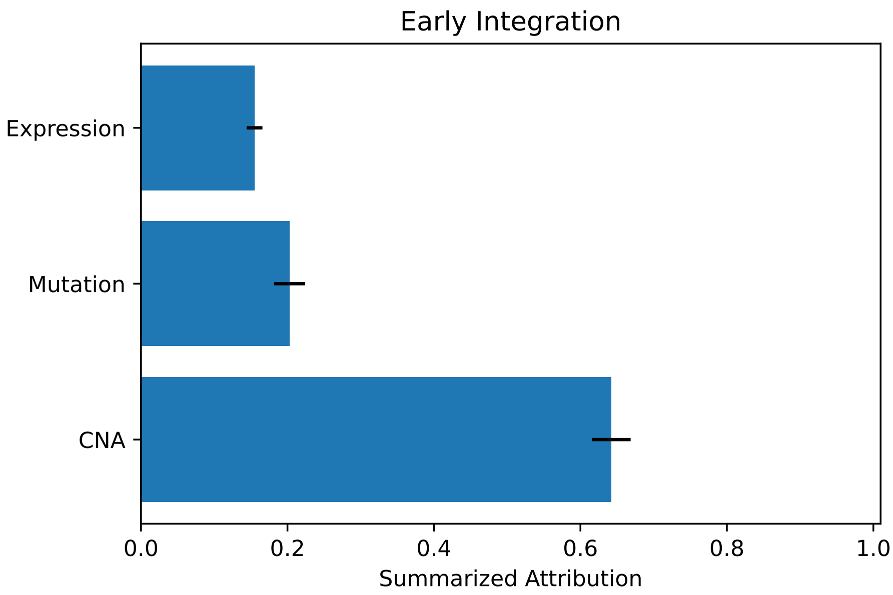

Figure 2: Mean and standard deviation over all drug data sets of each omics' summarized and normalized attributions for Early Integration.

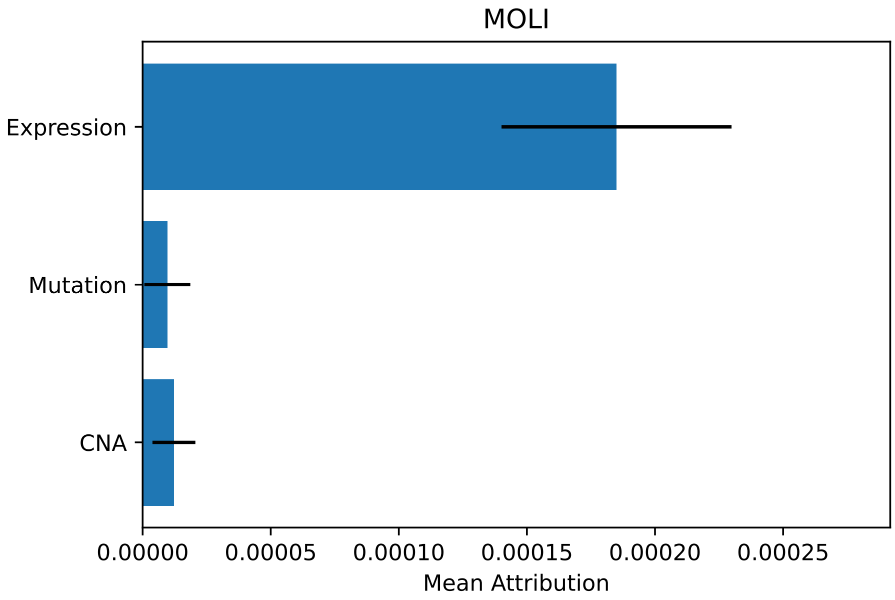

Figure 3: Mean and standard deviation over all drug data sets of each omics' mean attributions for MOLI.

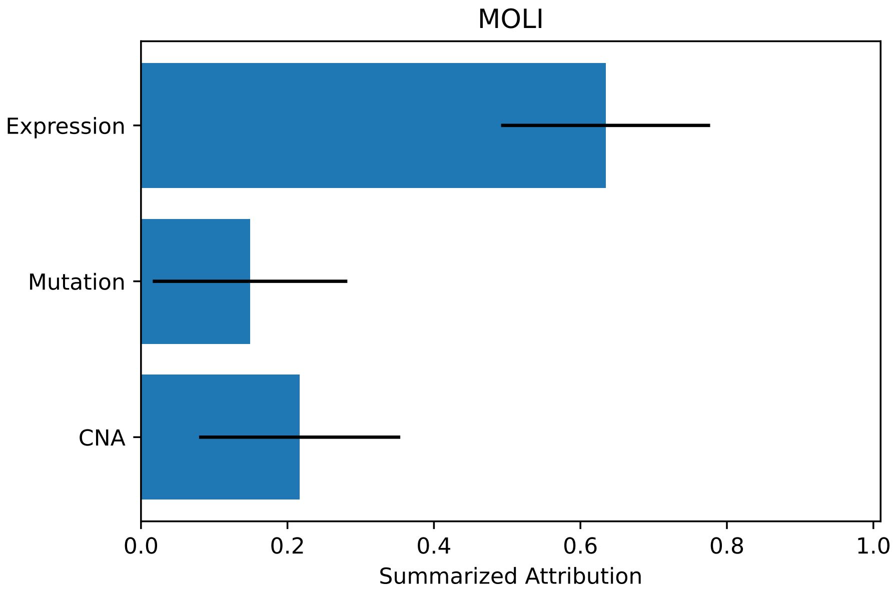

Figure 4: Mean and standard deviation over all drug data sets of each omics' summarized and normalized attributions for MOLI.

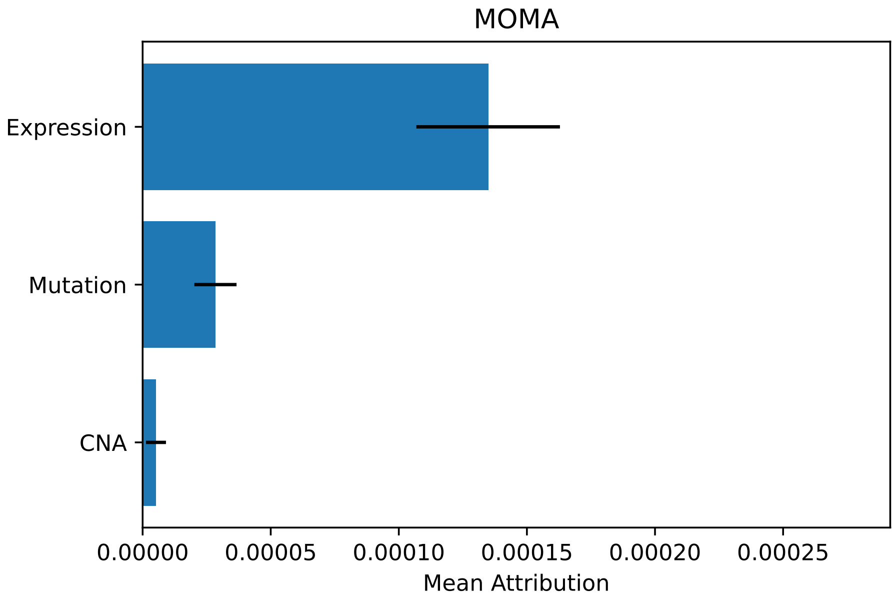

Figure 5: Mean and standard deviation over all drug data sets of each omics' mean attributions for MOMA.

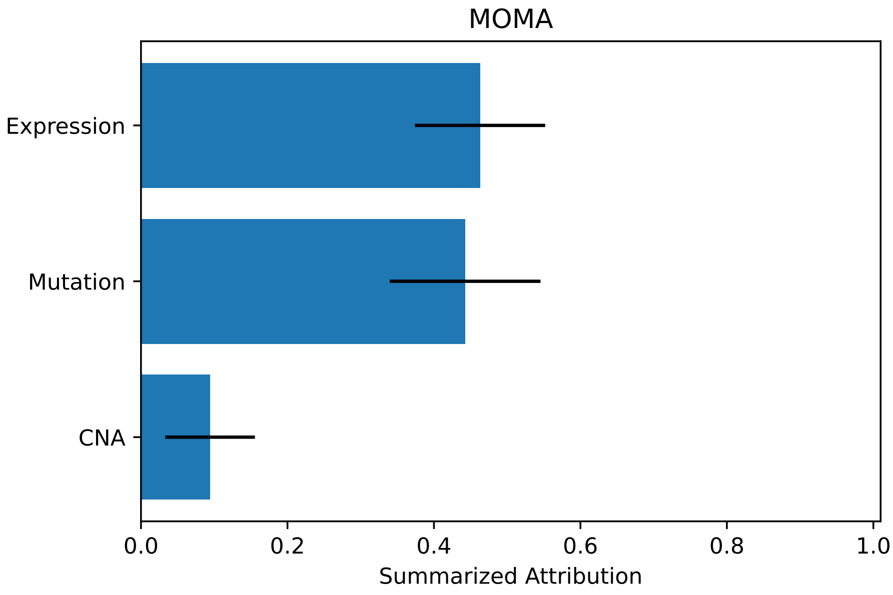

Figure 6: Mean and standard deviation over all drug data sets of each omics' summarized and normalized attributions for MOMA.

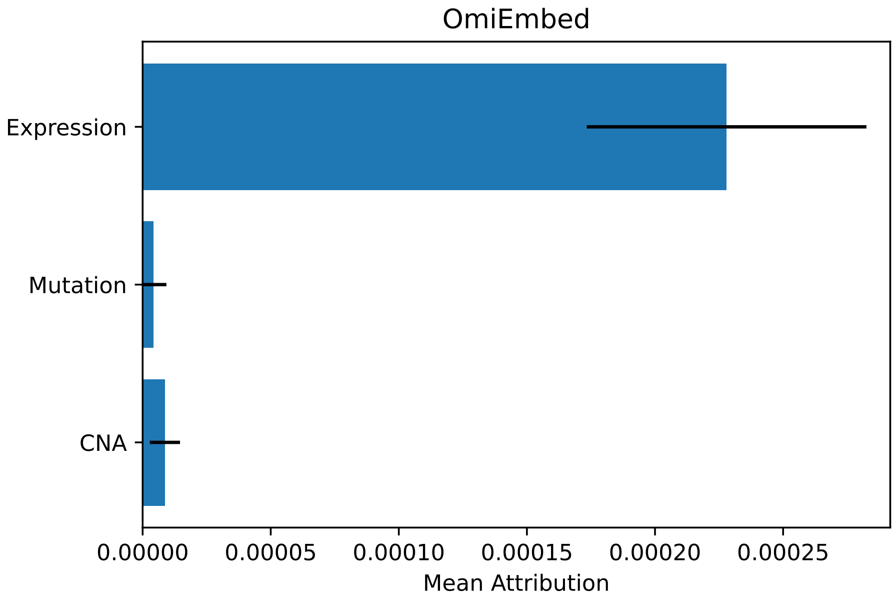

Figure 7: Mean and standard deviation over all drug data sets of each omics' mean attributions for OmiEmbed.

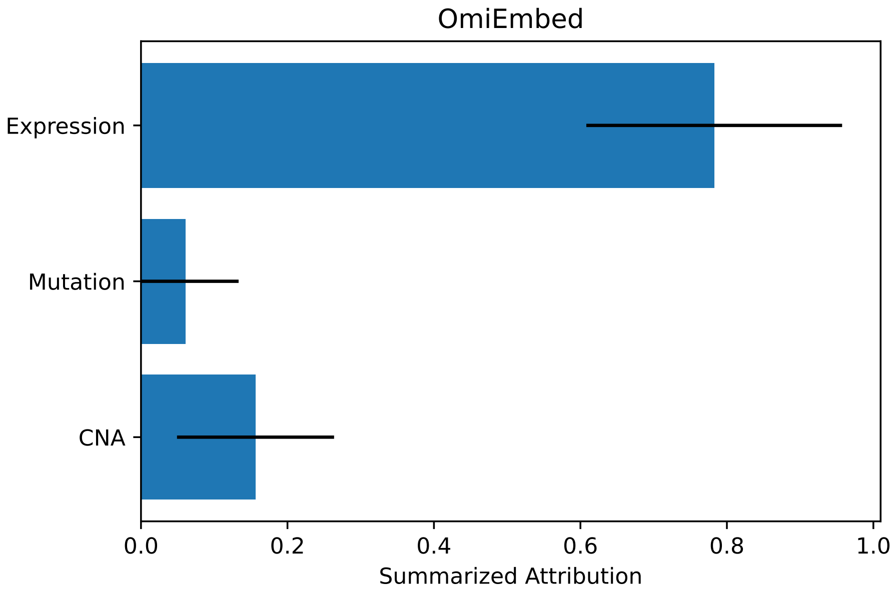

Figure 8: Mean and standard deviation over all drug data sets of each omics' summarized and normalized attributions for OmiEmbed.

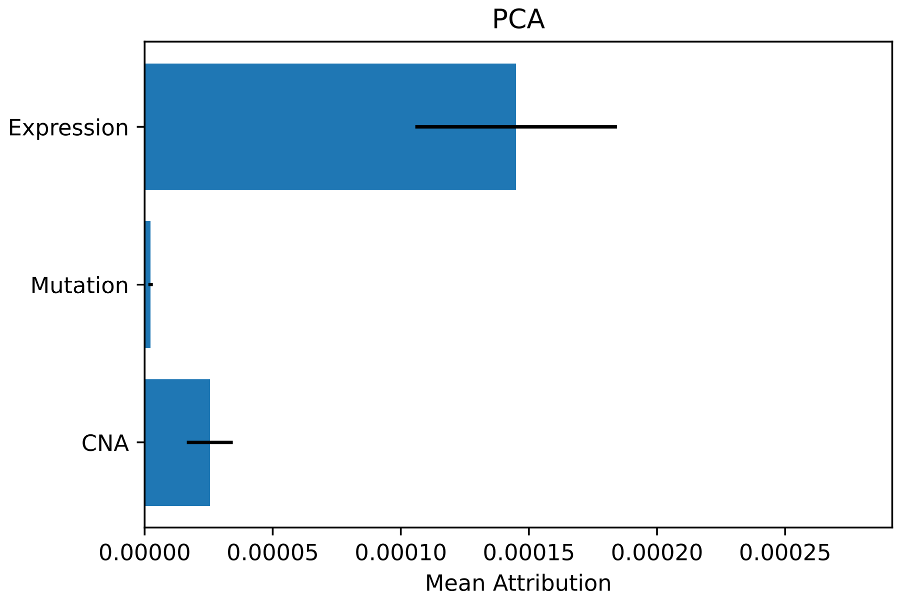

Figure 9: Mean and standard deviation over all drug data sets of each omics' mean attributions for PCA.

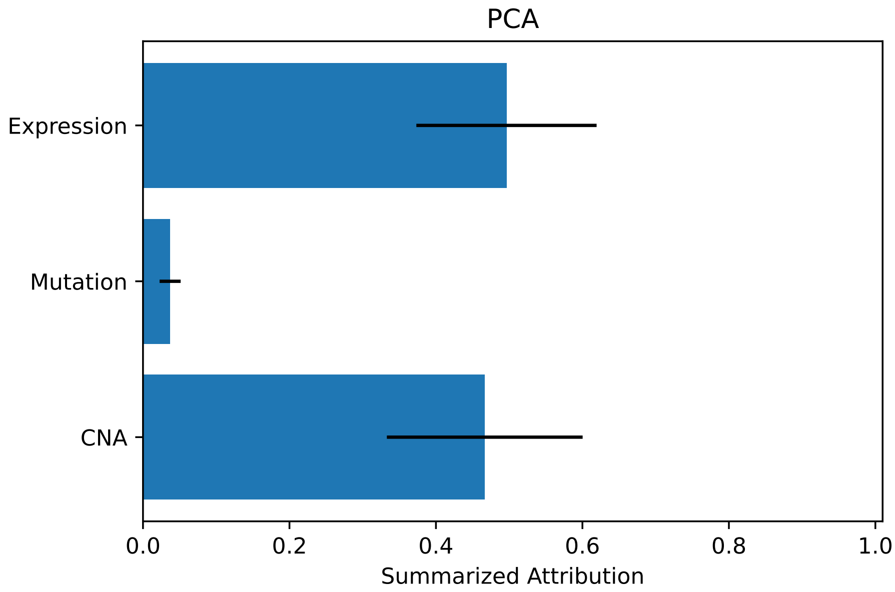

Figure 10: Mean and standard deviation over all drug data sets of each omics' summarized and normalized attributions for PCA.

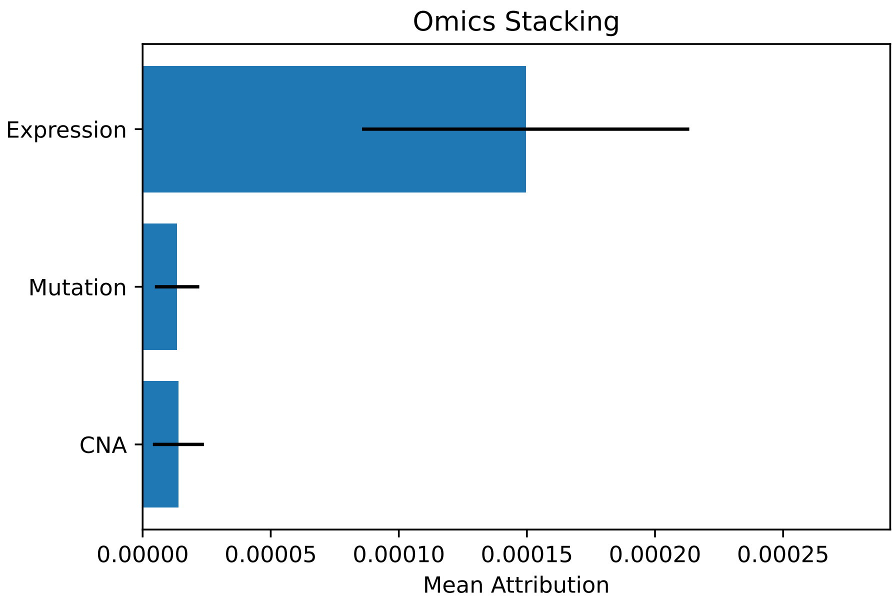

Figure 11: Mean and standard deviation over all drug data sets of each omics' mean attributions for Omics Stacking.

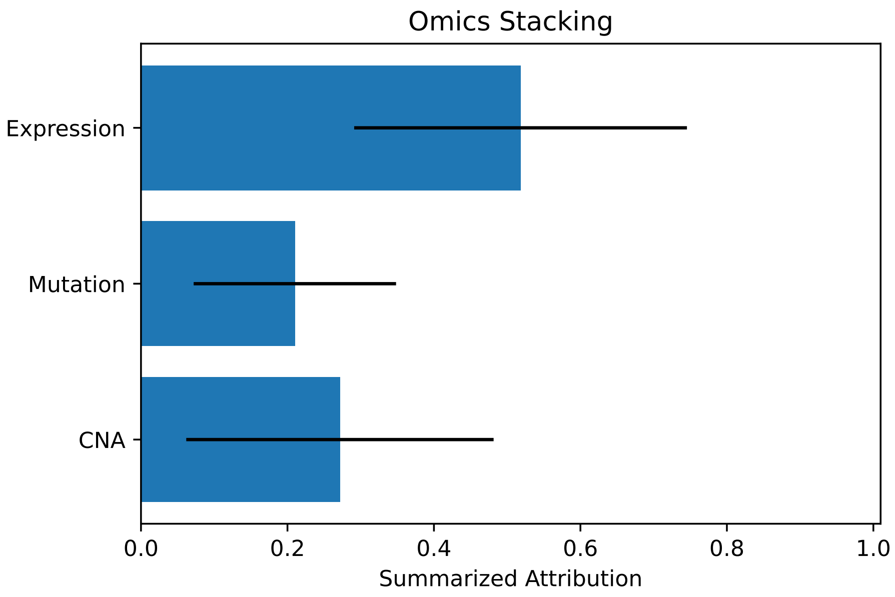

Figure 12: Mean and standard deviation over all drug data sets of each omics' summarized and normalized attributions for Omics Stacking.

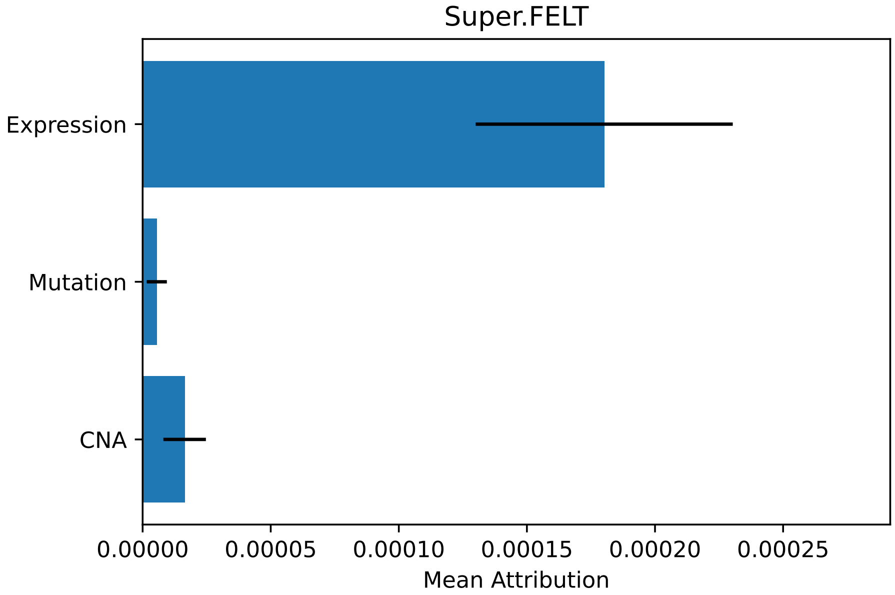

Figure 13: Mean and standard deviation over all drug data sets of each omics' mean attributions for Super.FELT.

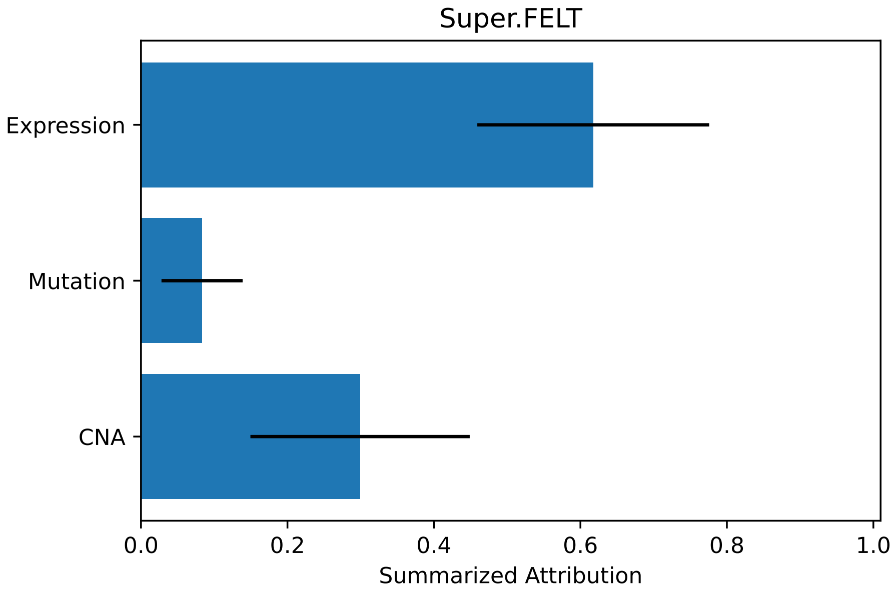

Figure 14: Mean and standard deviation over all drug data sets of each omics' summarized and normalized attributions for Super.FELT.
